# Sample Size Requirements of a Pharmaceutical Material Library: A Case in Predicting Direct Compression Tablet Tensile Strength by Latent Variable Modeling

**DOI:** 10.3390/pharmaceutics16020242

**Published:** 2024-02-07

**Authors:** Junjie Cao, Haoran Shen, Shuying Zhao, Xiao Ma, Liping Chen, Shengyun Dai, Bing Xu, Yanjiang Qiao

**Affiliations:** 1 Department of Chinese Medicine Informatics, School of Chinese Materia Medica, Beijing University of Chinese Medicine, No. 11, North Third Ring East Road, Beijing 100029, China; 20190941370@bucm.edu.cn (J.C.); 20190941374@bucm.edu.cn (H.S.); 20230935202@bucm.edu.cn (S.Z.); 20230935203@bucm.edu.cn (X.M.); 20230935129@bucm.edu.cn (L.C.); 2 Beijing Key Laboratory of Chinese Medicine Manufacturing Process Control and Quality Evaluation, Beijing 100029, China; 3 National Institutes for Food and Drug Control, Beijing 100050, China; daisy@nifdc.org.cn

**Keywords:** material library, sample size, few-shot modeling, compression behavior classification system (CBCS), tabletability, formulation design, hierarchical sampling, latent variable modeling, tablet tensile strength

## Abstract

The material library is an emerging, new data-driven approach for developing pharmaceutical process models. How many materials or samples should be involved in a particular application scenario is unclear, and the impact of sample size on process modeling is worth discussing. In this work, the direct compression process was taken as the research object, and the effects of different sample sizes of material libraries on partial least squares (PLS) modeling in the prediction of tablet tensile strength were investigated. A primary material library comprising 45 materials was built. Then, material subsets containing 5 × *i* (*i* = 1, 2, 3, …, 8) materials were sampled from the primary material library. Each subset underwent sampling 1000 times to analyze variations in model fitting performance. Both hierarchical sampling and random sampling were employed and compared, with hierarchical sampling implemented with the help of the tabletability classification index *d*. For each subset, modeling data were organized, incorporating 18 physical properties and tableting pressure as the independent variables and tablet tensile strength as the dependent variable. A series of chemometric indicators was used to assess model performance and find important materials for model training. It was found that the minimum *R*^2^ and *RMSE* values reached their maximum, and the corresponding values were kept almost unchanged when the sample sizes varied from 20 to 45. When the sample size was smaller than 15, the hierarchical sampling method was more reliable in avoiding low-quality few-shot PLS models than the random sampling method. Two important materials were identified as useful for building an initial material library. Overall, this work demonstrated that as the number of materials increased, the model’s reliability improved. It also highlighted the potential for effective few-shot modeling on a small material library by controlling its information richness.

## 1. Introduction

In pharmaceutical design and development, the material library or material database is an emerging, new, and efficient approach for organizing physical property data of materials like active pharmaceutical ingredients (APIs), excipients, or intermediates. The material library method aims to develop a standard material characterization framework to collect and store the physiochemical properties and related information of pharmaceutical materials [1]. By using a material library, both new and generic drug development activities could be enhanced in different ways, such as by understanding the relationship among different material quality attributes and simplifying the material characterization workload, finding the surrogate or equivalent materials for costly APIs during initial process development, and supporting the development of process models by linking the materials’ physical properties to unit operations [2,3,4,5,6,7].

In practice, the material library was presented in the form of tabular data, in which the rows were different materials, and the columns were material quality attributes. Table 1 shows the typical sizes of reported pharmaceutical material libraries after retrieving articles in Web of Science using “material library” or “material database” as key words [1,3,4,7,8,9,10,11,12,13,14,15,16,17,18,19,20,21,22,23]. The material name and attributes in each material library are shown in Table S1 in the Supplementary Materials. From the aspect of material attributes, it can be seen that the numbers of material property descriptors are mainly spread in the range of 8 to 55. In particular, Van et al. [8] built a material library involving over 100 raw material descriptors. The investigated material properties are usually function-related or process-oriented. For instance, Wang et al. [12] tried to predict feeder performance based on material flow properties. Generally speaking, a thorough evaluation of material properties is the prerequisite for building predictive models for in silico processes and formulation development. But this does not mean that more material property descriptors are better, since there may be inter-relationships among them and more descriptors mean a higher cost of characterization. Van et al. [8] proved that correlated descriptors in the raw material property database could be simplified using a multivariate data analysis (e.g., principal component analysis).

In addition, the requirements for sample size and sample diversity of a material library also need to be considered to obtain a high-quality dataset. As shown in Table 1, the sample sizes of most material libraries range from 10 to 130. Material types are often determined empirically by considering the following aspects: (1) selecting materials with different deformation behavior (e.g., plastic or brittle) [5,24,25]; (2) choosing materials with different chemical compositions like APIs, cellulose, lactose, starch, or calcium hydrogen phosphate [26,27,28]; (3) using different pharmaceutical excipients like fillers, binders, lubricants, or disintegrants [8,27]; (4) enriching material variability by incorporating materials from different suppliers or with different grades [29,30,31].

Conventionally, sufficiently more observations serving as a training set are considered favorable before modeling in machine learning. For instance, in the field of artificial neural networks, there is a set of rules of thumb regarding sample size requirements. (1) The sample size needs to be at least a factor 50 to 1000 times the number of prediction classes; (2) the sample size needs to be at least a factor 10 to 100 times the number of the features; (3) the sample size needs to be at least a factor 10 times the number of parameters (i.e., synaptic weights and biases) in the network [2,32]. In fact, collecting and annotating high-volume data meeting these above requirements is time-consuming and expensive. To overcome this obstacle, some research has discussed the feasibility of minimum dataset size and few-shot learning, which does not result in significant model performance loss in the presence of limited data. Bongiorno et al. [33] constructed sample sets ranging from 10 to 50,000 to study the effect of dataset size on model training performance and found that approximately 200 examples were generally sufficient to train a machine learning algorithm, and increasing the number of training samples did not significantly improve the accuracy of the results. Li et al. [34] proposed an indicator $g^{2}$, which was used to assess the model structure to analyze the minimum size of data to construct a valid model. The verification found that with the increase in the number of samples of the modeling dataset, the model became stable, as the $g^{2}$ index converged to zero. Althnian et al. [35] found that the overall performance of classifiers depended on how well a dataset represented the original distribution rather than its size. These studies demonstrated that it was possible to find the suitable sample size for modeling purposes. As far as we know, the impacts of sample size in the material library domain on data-driven modeling have not been studied.

Direct compression (DC) is a desirable tablet manufacturing route because of fewer unit operations, shorter operating time, and lower labor costs [30,31,36]. In our previous work [4], the tabletability index *d* was proposed to differentiate five categories of tensile strength (*TS*) vs. pressure (*P*) relationships. If the index *d* is higher than 0.5, this material falls into Category 1, indicating excellent tabletability at the low-pressure range. If a material belongs to Category 2A or 2B, the compression force needs to be fine-tuned when material is compressed. When a material belongs to Category 2C or 3, the material requires special attention, as it may exhibit poor tabletability at most pressures. In this paper, both the number and type of samples when constructing the material library are investigated. A primary material library containing 45 fully characterized pharmaceutical materials was constructed. Different subsets of the primary material library were sampled to simulate effects of changes in sample size, and these subsets were applied to construct DC process models. At the given sample size of each subset, the supervised sampling method was performed by the tabletability index *d* to control sample diversity, and was compared with the totally random sampling method. The minimum material library requirements in terms of size and type were estimated by analyzing the performance of DC process models. The results of this study would be useful guides for selecting materials and organizing a diversified small-size material library, resulting in shorter material accumulation time as well as quicker process model development.

## 2. Experimental Methods

### 2.1. Construction of the Primary Material Library

A total of 45 powdered materials, including 32 pharmaceutical excipients and 13 natural production powders (NPPs), were carefully selected from a homemade database named intelligent TCM (iTCM) [4,37]. Different batches or types of the same material, exhibiting different capacities, were considered in the material library. For instance, seven types of MCC powders, including PH102, PH200NF, Oricel™PH-102 SCG, Oricel™PH302NF, Oricel™PH-112, Oricel™PH302NF, and vivapur^®^ type200, were included. These 45 samples were used as the primary material library and were divided into 5 categories (Cat.1, 2A, 2B, 2C, 3; Cat.1 denotes Category 1, and the same nomenclature applies to the others) by the tabletability index *d*. Each category included 9 samples. The names, lot numbers, and suppliers for all materials are described in Table S2 in the Supplementary Materials. All 45 powdered materials are described by 27 quality attributes, including 18 physical powder parameters and 9 compression descriptors, as outlined in Table 2.

Among the 27 powder properties, 12 parameters were measured or calculated by standard testing procedures of the SeDeM expert system methodology [38,39]. These parameters include bulk density (*ρ*_b_, g·cm^−3^), tapped density (*ρ*_t_, g·cm^−3^), inter-particle porosity (*Ie*), Carr’s index (*IC*), Hausner ratio (*IH*), angle of repose (*AOR*, °), flow time (*t*″, s), cohesion index (*Icd*, N), loss on drying (*HR*%), hygroscopicity (*H*%), proportion of particles smaller than 50 μm (%*Pf*), and homogeneity index (*Iθ*). The dimensions of powder can be expressed by *ρ*_b_ and *ρ*_t_. The parameters *IC*, *Ie,* and *Icd* characterized the compressibility of powders. Descriptors *AOR*, *t*″, and *IH* reflect the flowability of powder. The stability of powder can be described by the parameters *HR%* and *H%*. Physical properties %*Pf* and *Iθ* represent the uniformity of the powder. The remaining 6 physical properties include true density (*D*_t_, g·cm^−3^), particle sizes (i.e., *D*_10_, *D*_50_, and *D*_90_, μm), particle size distribution width (Span), and solid fraction (SFp). The compression curve (*TS* vs. pressure *P*) data for each material were also stored in the iTCM database. As for compression descriptors, different compression equations are used, respectively, to interpret the compressibility, compactability, and tabletability. The compressibility of a powder is the powder’s ability to deform under pressure, and it is described by the indexes of *Kawakita a*, *ab,* and *b*^−1^, *Heckel P*_y_, *Shapiro f*, and *Gurnham K* [40,41,42]. The compactability of a powder is the ability to form mechanically strong compacts and is expressed by *Ryshkewitch–Duckworth k*_b_ [41,42,43]. The tabletability of powders, defined as the capacity of a powdered material to be transformed into a tablet of sufficient strength under the prescribed pressures, can be indicated by the index of *Power d* [4,43,44].

### 2.2. Construction of Material Libraries with Different Sample Sizes

**Figure 1:**
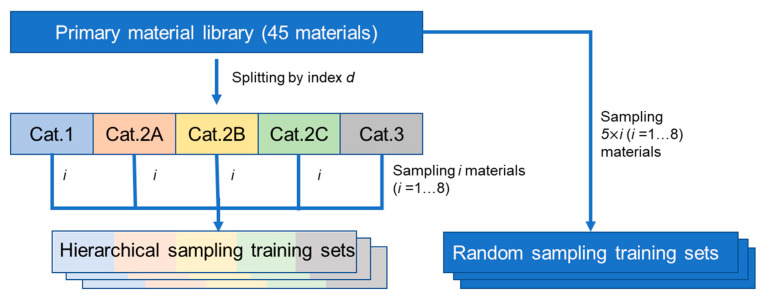
The schematic diagram of two sampling methods. Cat.1 is the abbreviation of Category 1, and the same goes for the rest.

To investigate the influence of different sampling methods on the prediction performance of the model, a supervised sampling method under the guidance of index *d* and a random sampling method were used to construct training datasets from the primary material library. The sampling procedures are shown in Figure 1. As for the supervised sampling method, *i* (*i* represents the number of sampled materials from the primary library; *i* = 1, 2, 3, …, 8) materials were systematically selected from the 5 categories in turn and then merged to construct the training dataset with different sample sizes. To analyze the variation in model fitting performance with different numbers of samples, each training pattern was sampled 1000 times under a given *i* condition. Consequently, 8 groups of hierarchical sampling training datasets (HST*i*) were obtained. Regarding the random sampling method, 5 × *i* (*i* = 1, 2, 3, …, 8) materials were randomly sampled from the material library. Similar to the hierarchical sampling, each training pattern was sampled 1000 times under a given *i* condition and 8 groups of random sampling training datasets (RST*i*) were obtained. The dataset containing all 45 materials was also used as the training set and denoted as HST9 and RST9, respectively. The sampling program was compiled on the Matlab R2019a platform (Mathworks, Natick, MA, USA).

### 2.3. Predictive Modeling and Model Evaluation

The material property data matrix for the primary material library was organized to contain 27 columns and 45 rows. Similarly, the material property data matrix for the sampled material library was organized to contain 27 columns and 5 × *i* (*i* = 1, 2, 3, …, 9) rows, with the rows representing different materials and the columns representing material quality attributes. These quality attributes comprise 27 parameters, encompassing 18 physical powder parameters and 9 compression descriptors. The principal component analysis (PCA) was performed to compress the number of correlated material property variables into a smaller number of uncorrelated variables called principal components (PCs). PCs are ranked from the highest to the lowest variance. The score plot shows sample locations in the PC space, facilitating the detection of sample patterns and grouping similarities and differences. The loading plot helps interpret the relationships between the variables [45]. Before modeling, the data were scaled and centered. The PCA analysis was performed using SIMCA 13.0 (Umetrics, Umea, Sweden) software.

The partial least squares (PLS) regression method is employed to reduce the input dataset to a set of latent variables, which are linear combinations of the original variables. PLS assesses the relationship between the input space ($X\in R^{n\times m}$, where *n* and *m* represent the sample and the number of variables in turn) and the output space (Y$\in R^{n\times l}$, where *l* is the number of output variables). The decomposition occurs in the feature, where the score matrix, load matrix, and latent variables (LVs) are calculated [46]. Additionally, the goodness-of-fit indexes (i.e., *R*^2^*X* and *R*^2^*Y*) are associated with the amount of variability captured by the LVs in PLS analysis. The goodness of prediction is estimated by 10-fold cross-validation method. In this work, each sample in the material property data matrix was combined with a set of pressure (*P*) vectors. Each tablet tensile strength (*TS)* corresponded to a pressure value in the compression curve. The DC compression dataset of the primary material library was constructed with 1090 rows and 20 columns, comprising 18 physical properties and the tableting pressure as the independent variables and *TS* as the dependent variable. *Z*-score was used to standardize and normalize the 19 independent variables. The first 4 LVs explained (*R*^2^*X*) 71.9% and (*R*^2^*Y*) 88.1% of the variability of 45 samples. Adding one more latent factor did not enhance the model performance, and thus, 4 latent variables in the PLS analysis were set. The PLS algorithms were performed on the Matlab 2019a software (Mathworks, Natick, MA, USA) with the PLS Toolbox 2.1 (Eigenvector Research Inc., Wenatchee, WA, USA).

These few-shot HST (RST) models, constructed in Section 2.2, were validated by 10-fold cross-validation and external validation to evaluate the predictive ability of the constructed model. The indicators used for predictive model evaluation include the coefficient of determination ($R^{2}$) and the root mean square error (*RMSE*), the coefficient of determination at validation set ($R^{2}p$), and the root mean square error at validation set (*RMSEp*). The *SCORE* parameter, representing the ratio of the correlation coefficient to the mean absolute error percentage (*MAPE*), was employed. A higher *SCORE* value indicates better prediction performance of the model. The *SCORE* parameter was specifically used to extract the model for subsequent important material analysis. The *MAPE* value, which measures the error between prediction and observation in regression analysis and model evaluation, is employed to eliminate the variable unit compared to the *RMSE* [47]. The model evaluation indexes mentioned above are defined as follows in Equations (1)–(4).

(1) $$R^{2}={\left(\frac{Cov\left(Y_{obs},Y_{pre}\right)}{\sigma_{obs}\times \sigma_{pre}}\right)}^{2}$$

(2) $$RMSE=\sqrt{\frac{\sum_{i=1}^{N}{\left(Y_{i}^{obs}-Y_{i}^{pre}\right)}^{2}}{n}}$$

(3) $$SCORE=\frac{R^{2}}{MAPE}$$

(4) $$MAPE=\frac{1}{n}\sum_{i=1}^{n}\left|\frac{Y_{i}^{obs}-Y_{i}^{pre}}{Y_{i}^{obs}}\right|$$

**Figure 2:**
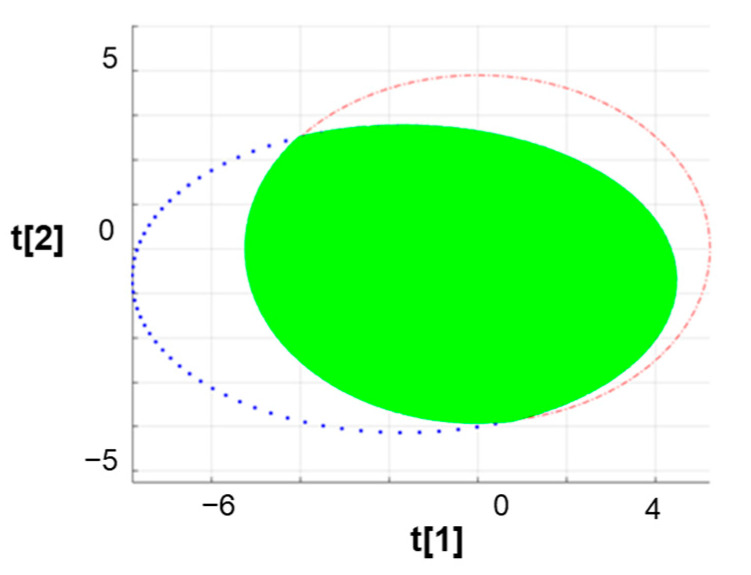
The 95% confidence ellipse in the PCA model. The red ellipse represents all 45 materials. The blue ellipse represents the sampled materials. The green area represents the overlapping area.

In addition to the commonly used chemometric indicators, a new method for evaluating the sample diversity of a selected material library has been proposed. The 95% confidence ellipse of the two-dimensional PCA score data is used to visualize the degree of aggregation of the data, and the eigenvalues and eigenvectors of the first two principal components are obtained [48]. The overlapping area between the confidence ellipse of sampled materials and the confidence ellipse of the primary material library samples is then calculated. Subsequently, the percentage of the overlapping area compared to the confidence ellipse area of the primary material library is obtained (Equation (5)). There is an illustrative diagram for calculating the index of the overlapping area (Figure 2). The calculation support was carried out on Matlab (R2019a).

(5) $$The\;overlapping\;rate=\frac{overlapping\;area\;}{95\%\;ellipse\;area\;of\;all\;samples\;}\times 100\%$$

### 2.4. Establishment of the External Validation Set

The external validation set was designed to test the prediction performance of established training models. Mixtures in the validation set were obtained using the full factorial design including three factors, i.e., Composition A, Composition B, and the ratio of A to B, as shown in Table 3. Composition A has two levels, representing two pharmaceutical excipients (i.e., microcrystalline cellulose PH102, lactose Tablettose^®^ 80). The MCC PH102 and lactose Tablettose^®^ 80 are two commonly used diluents in tablet formulation, and they represent the plastic and brittle compaction behaviors, respectively [49,50,51]. MCC PH102 was classified into Category 1 tabletability, and lactose Tablettose^®^ 80 was classified into Category 2B by the index *d*. Composition B has four levels, representing 4 natural production powders (i.e., *Stellariae Radix* extract, *Radix Rehmanniae* Preparata extract, *Rhizoma Alismatis* extract, and *Flos Farfarae* extract). The 4 NPPs were independent with 45 materials and were classified into Category 2A tabletability. The ratio of A to B is a continuous variable, and three levels are designed as 1:3, 1:2, and 1:2 in *w*/*w*, respectively. As a result, 24 formulations were generated from the design. Different mixtures were expected to exhibit different compaction behaviors, serving the validation purpose.

Each binary blend was mixed in a three-dimensional mixer for 10 min. A total of 0.5% magnesium stearate was added and mixed for an additional 5 min. The blended powders were then compressed into tablets using a single punch tablet press machine (C&C600A, Beijing C&C CAMBCAVI Co., Ltd., Beijing, China) equipped with a flat-faced punch and die with a 10 mm diameter. The magnesium stearate was used to lubricate the punch surfaces and the die walls before each compaction. After lubrication, the powders were manually filled into the die. Considering the different bulk densities of the materials, the filling mass was set to 300 mg or 350 mg to ensure the smooth ejection of the tablet. For each material, 3 compression pressures (5, 7, and 9 KN, where 1 KN = 12.74 Mpa) were applied to produce tablets with varying hardness. The applied mean velocity of the upper punch was 28 mm/s. At least three tablets were obtained under each pressure. The prepared tablets were sealed in a ziplock bag. After being stored for 24 h, the weight (GL124-1SCN, Beijing Sanfu Hezhong Technology Development Co., Ltd., Beijing, China), diameter, thickness (547-401 Digimatic Caliper, Mitutoyo, Kawasaki city, Japan), and diametrical crushing force (YPD-500, Shanghai Huanghai medicine inspection instrument Co., Ltd., Shanghai, China) of the tablets were measured. The tensile strength (*TS*) of the tablet was calculated as follows (Equation (6)).

(6) $$TS=\frac{2F}{\pi DH}$$

where *F* (N) is the tablet crushing force, *D* (mm) is the tablet diameter, and *H* (mm) is the tablet thickness [52].

The physical characterizations of single validation materials were published previously [4], and the physical properties of the binary mixture were calculated using the ideal mixing rule (Equation (7)). The physical properties of every single material were multiplied by its ratio and summed. For the external validation set, a total of 72 data records were obtained.

(7) $${property}_{mix}=\sum {ratio}_{i}\times {property}_{i}$$

## 3. Results and Discussion

### 3.1. The Powder Properties

Forty-five materials were divided into five categories according to the tabletability index *d*, and the boxplots of bulk density, cohesion, angle of repose, and median particle size (*D*_50_) are shown in Figure S1 in the Supplementary Materials. Boxplots provide a visual representation of data distribution, including showing the minimum, first quartile (Q1), median, third quartile (Q3), and maximum. The bulk density (*ρ*_b_) across the 45 batches of powders was wide, ranging from the very lightly packed MCC vivapur^®^ type 102 (0.31 g·cm^−3^, Cat.1) to the densely packed *Radix Polygoni Multiflori* semi-extract powder (0.71 g·cm^−3^, Cat.3). Among the five types of materials, Cat.1 materials exhibited the smallest average bulk density (0.36 g·cm^−3^), while Cat.2C materials had the largest bulk density (0.62 g·cm^−3^). The cohesion index (*Icd*), directly proportional to the compaction of powder, changed from 9.7 N (calcium hydrogen phosphate, Cat.2C) to 366.8 N (MCC PH102, Cat.1) [53]. The cohesion index was consistent with the results of the five subcategories. The angle of repose (*AOR*) is directly reflected in the flowability of the powdered material, which is related to inter-particulate friction or resistance to movement between particles. According to the USP-NF<1174>, if the *AOR* is less than 45°, the powder could flow in such a way to meet industrial production requirements with or without aid. But if it exceeds 50°, the flow is rarely acceptable for manufacturing purposes. Angle of repose values in this research varied between 31.8° (Flowlac^®^ 100) and 57.3° (Granulac^®^ 200). The mean values of *AOR* in Cat.1, 2A, 2B, 2C, and 3 were 40.6, 43.7, 45.0, 47.5, and 49.0, respectively. The *D*_50_ values varied greatly between different powders, from 12.6 μm to 253.6 μm. The *D*_50_ values of Cat.1 materials were larger than 100 μm, except MCC PH102NF (94.2 μm) and Ethyl Cellulose N-7 Pharm (65.5 μm). In Cat.2 and Cat.3, the *D*_50_ values of 31 materials were no more than 100 μm. Overall, Cat.1 materials generally had the smallest bulk densities, the strongest cohesion, the smallest angle of repose, and the largest median particle sizes. The *Icd* and *AOR* parameters for Cat.2B materials showed the widest distribution range. The properties of Cat. 2C and Cat. 3 materials were similar.

**Figure 3:**
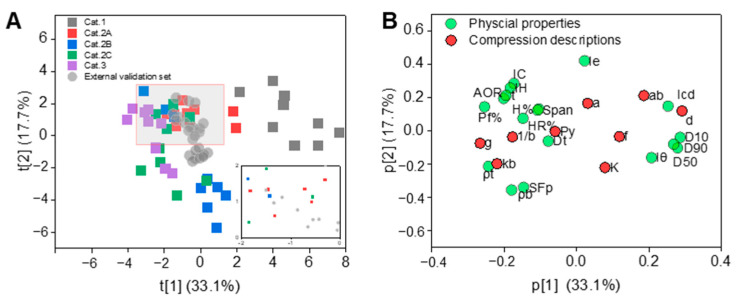
(**A**) The PCA score plot of 45 materials. (**B**) The PCA loading plot of 27 variables. The grey square represents Category 1 tabletability. The red square represents Category 2A tabletability. The blue square represents Category 2B tabletability. The green square represents Category 2C tabletability. The purple square represents Category 3 tabletability. The grey circle represents external validation set. The green circle represents the physical property of materials. The red circle represents the compression descriptor of materials.

Furthermore, the data matrix of the primary material library was organized to contain 27 columns and 45 rows. The PCA model projected the 27 variables to a latent space with four PCs. The first two PCs explained (*R*^2^*X*) 50.9% and predicted (*Q*^2^*X*) 36.5% of the variability of the data. The score plot (Figure 3A) and the loading plot (Figure 3B) for the first two PCs were generated. In the score plot, the 45 samples in the primary material library were colored based on five tabletability categories. The loading information revealed that compression descriptions *d*, *g,* and *k*_b_ were mainly associated with PC1. Parameters *a* and *K* were mainly associated with PC2. The Cat.2C and Cat.3 materials overlapped, since both of them had poor tabletability. Combined with the loading plot, it was found that the Cat.2C and Cat.3 materials were opposite to the position of particle size parameters (*D*_10_, *D*_50_, *D*_90_, *Iθ*), and had the same position in terms of *AOR*, %*Pf,* and *ρ*_t_. The Cat.1 materials were relatively concentrated in the positive half-axis and were opposite to the Cat.2C and Cat.3 materials. Cat.2A materials were concentrated in the center of the score plot, indicating that they had moderate material properties. Cat.1 materials had lower *P*_y_ and *K* values than Cat.2A materials, revealing that the former could be compressed easily [54]. The data for 24 binary powders in the external validation set were also projected onto the score plot. It could be seen that the mixtures were spread within the region of Cat.1, Cat.2A, and Cat.2B materials, from which two of the mixtures were made.

### 3.2. Comparison of Model Performance

Nine groups of HST and RST models were constructed and evaluated using the methods mentioned in Section 2.2 and 2.3. An external validation set, as described in Section 2.4, was employed to evaluate the prediction performance of the HST and RST models. The values for $R^{2}$**,** *RMSE*, $R^{2}p$, and *RMSEp* in each group of the HST (RST) models were calculated and recorded.

#### 3.2.1. The $R^{2}$ Values during Cross-Validation

**Figure 4:**
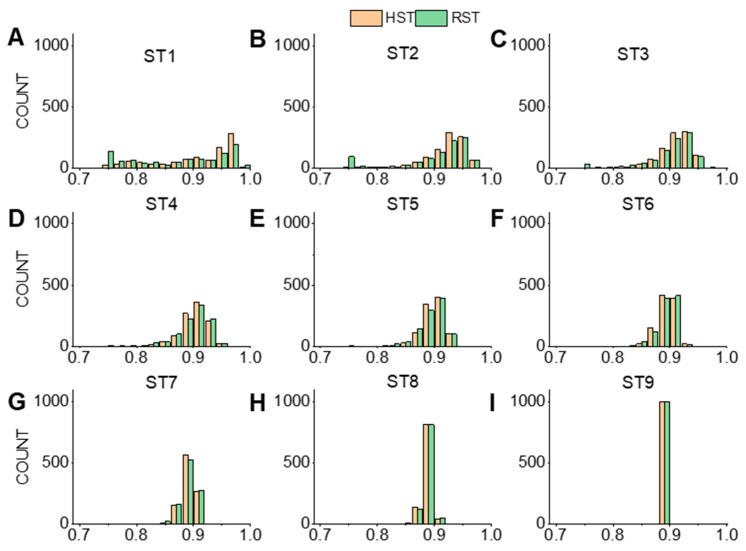
The histograms of correlation coefficients from cross-validation. Each subgraph (**A**–**I**) represents a group of few-shot sampling training dataset ST*i* models (sample size = 5 *× i, i* = 1~9). The orange color represents hierarchical sampling models and the green color represents random sampling models.

The mean, peak width, and extreme values of $R^{2}$ in each group of 1000 sampling results were calculated. The extreme values, i.e., the maximum (Max) and the minimum (Min) $R^{2}\;$values for each group, are shown in Table S3 in the Supplementary Materials. The frequency distribution histograms are drawn (Figure 4). The peak width was obtained by fitting the frequency distribution results with a Gaussian function.

When the number of materials was 5 (*i* = 1, corresponding to modeling data sizes in the range of 63~190 rows), the peak width of RST1 was 0.57, while that of HST1 was 0.46, with the latter being smaller than that of the RST model. From ST2, the peak width of RST was nearly equal to that of HST, and the peak width of ST ranged between 0.01 and 0.05. The mean $R^{2}$ values for nine groups of HST models were stable in the range of 0.88~0.91. The mean $R^{2}$ values for nine groups of RST models were spread in the range of 0.86~0.88. The maximum $R^{2}$ values for each group under the two sampling methods were close, but the minimum $R^{2}$ values of the HST models were larger than those of the RST models. When the number of samples was less than 15 (*i* = 3, corresponding to modeling data sizes in the range of 243~482 rows), the frequency histograms of $R^{2}$ in HST models were more concentrated than that of RST models. This suggested that it was possible to avoid developing a model with poor fitting performance when the size of the material library was less than 15. When the number of samples exceeded 15, the $R^{2}$ values of HST and RST obtained became close, suggesting that the structure of the training set sample began to stabilize.

#### 3.2.2. The RMSE Values during Cross-Validation

The peak width and the extreme values of *RMSE* in each group of 1000 sampling results were calculated. The maximum and minimum *RMSE* values of the sampling dataset, the group of models ST1~ST9, were calculated, as shown in Table 4. The frequency histograms are shown in Figure S2 in the Supplementary Materials. It was observed that with the increase in the number of samples, the *RMSE* frequency histogram of the HST models and RST models tended to concentrate on the right side of the axis.

When the number of materials was 5 (*i* = 1), the peak width of RST1 was 0.41 and the peak width of HST1 was 0.33. From the sample size 15 (*i* = 3, corresponding to modeling data sizes in the range of 243~482 rows), the gap between HST peak width and RST became less than 0.1, indicating that the peak shape was concentrated. Comparing the maximum values of the models from ST1 to ST8, the differences between the HST models and RST models were less than 0.1. The minimum values of *RSME* in the eight groups of HST models were larger than those of RST. The maximum *RMSE* values of each group under the two sampling methods were closer, but the minimum *RMSE* values of the HST models were larger than those of RST. Comparing the *RSME* range of each group of models, it was found that the ranges of the remaining HST models were narrower than those of RST, especially for the HST1~3 models. The result was consistent with the $R^{2}$ results in Section 3.2.1.

#### 3.2.3. The $R^{2}p$ Values during External Validation

The mean, peak width, and extreme values of $R^{2}p$ in each group of 1000 sampling results were calculated. The extreme values, i.e., the maximum and the minimum $R^{2}p\;$values of each group, are shown in Table 5. The frequency distribution histograms are shown (Figure S3 in the Supplementary Materials). The peak width was obtained by fitting the frequency distribution results with a Gaussian function.

The peak width values of the HST2~8 models were larger than those of RST2~8 models, and the difference values between HST2~8 and RST2~8 were less than 0.01. The mean $R^{2}p$ values gradually increased as the number of sampling materials increased. The mean $R^{2}p$ values for the top three groups of RST models were spread in the range of 0.54~0.86. The mean $R^{2}p$ values for the top three groups of HST models were spread in the range of 0.62~0.87. The mean $R^{2}p$ values for the remaining six groups of HST models were spread in the range of 0.90~0.93, which was consistent with the range observed for the RST models. When the number of materials was 15, the minimum $R^{2}p$ value was 0.33 in the HST3 models, while the minimum $R^{2}p$ value in the RST3 models was 0.16. Comparing the extreme values of the HST and RST models, it was found that the minimum $R^{2}p$ values of the HST models were larger than those of the RST models, and the maximum $R^{2}p$ values of the HST models were similar to those of the RST models. As the numbers of materials increased, the prediction performance of the two sampling methods became similar. The HST models could be used for their superior performance, to avoid models with poor prediction performance, especially when the number of samples was smaller than 15.

#### 3.2.4. The RMSEp Values during External Validation

The mean, peak width, and extreme values of *RMSEp* in each group of 1000 sampling results were calculated. The extreme RMSEp values of each group are shown in Table 6. The frequency distribution histograms are shown (Figure S4 in the Supplementary Materials). The peak width was obtained by fitting the frequency distribution results with a Gaussian function.

The peak width values of *RMSEp* gradually decreased as the number of sampling materials increased. For HST1 models, the peak width of *RMSEp* was 1.48, compared to 1.67 for RST1 models. The peak width values of *RMSEp* for the HST4~9 models were similar to those of the RST6~9 models, and the differences between HST4~9 and RST 4~9 were less than 0.03. The mean *RMSEp* value of the HST1 models was 1.81, while that for the RST1 models was 2.08. The mean *RMSEp* values gradually decreased until the number of sampling materials reached 15 (*I* = 3, ST3). The differences in mean *RMSEp* values between the HST4~9 models and RST4~9 models were less than 0.03. The maximum *RMSEp* values were quite different. Almost all maximum *RMSEp* values of the RST models were larger than those of the HST models, especially for HST1 and RST1 (with a sampling number of 5). The maximum *RMSEp* of the HST1 models was 9.7, while that of the RST1 models was 41.56. The minimum *RMSEp* values in the HST and RST models were almost the same. It suggested that the differences in *RMSEp* values within or between ST models decreased, and the model prediction performance was gradually improved as the number of samples increased. When the number of samples was smaller than 15, the HST models were more effective for avoiding models with excessive errors and providing better generalization ability.

### 3.3. The Overlapping Rate of Confidence Area

**Figure 5:**
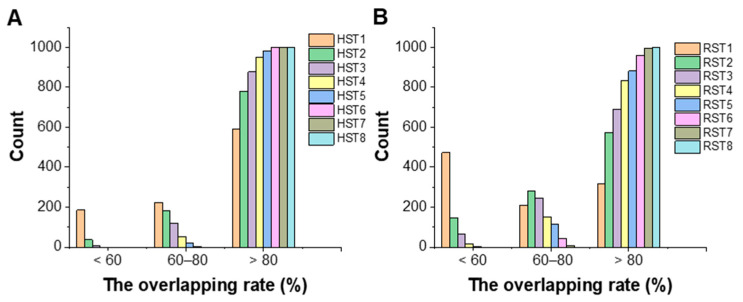
The histograms of index of overlapping area in 8 groups of few-shot models. (**A**) Hierarchical sampling models; (**B**) random sampling models.

The values for the index of overlapping area for eight groups of HST (RST) models were calculated according to Equation (5). The frequency histograms of overlapping area rate are drawn in three intervals of <60%, 60–80%, and >80%, respectively (Figure 5).

It was found that all the maximum overlapping area rates in the HST and RST models approached 100%, but the minimum value in the HST models was larger than that in the RST models. The total number of models with an overlap rate less than 60% in the HST models was smaller than that in the RST models. In particular, when the number of materials involved in a model was 5, the frequency of the overlapping area rate of the RST1 in the <60% interval exceeded 50%, while the corresponding frequency for the HST1 model was 24.4%. When the sampling number was 20 (i.e., HST4, RST4), the proportion of models with an overlapping area rate higher than 80% in the HST models was 94.8%, while that of the RST models was 83.3%. As the number of sampling materials increased, the overlapping area rate between the HST5 and RST5 models exceeded 90%, and the differences in overlapping area rate between the HST5 and RST5 models were gradually narrowed. To sum up, it was suggested that the hierarchical sampling was helpful for ensuring the diversity of samples. A value of 15 or 20 was a potentially acceptable number for sampling materials to construct a material library for few-shot modeling, where the material properties were closer to the population.

### 3.4. Finding Important Materials for Model Training

**Figure 6:**
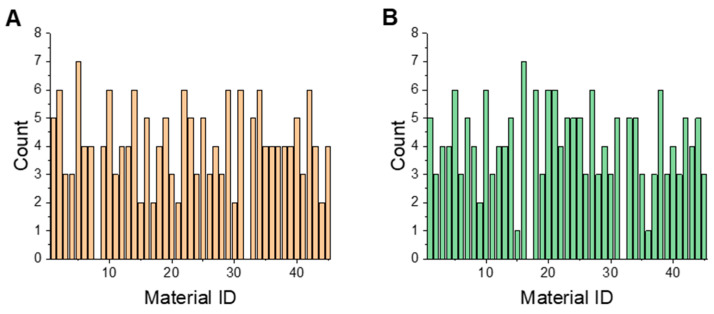
The histograms of potential important materials contained in (**A**) hierarchical sampling models with the maximum *SCORE* values; (**B**) random sampling models with the maximum *SCORE* values.

Finding important materials is useful for building a representative material library and training high-performance process models. These materials were identified by analyzing the occurrence frequency of materials in HST and RST models with high correlation coefficient and small prediction error. The model performance index *SCORE* parameter was proposed to screen high-performance models. The *SCOREcv* and *SCOREp* values for each group of HST (RST) models were calculated according to Equation (3), respectively. Then, the model with the maximum *SCORE* value (=*SCOREcv* + *SCOREp*) from the 1000 models in each group of HST*i* or RST*i* models was found. The materials used in the identified models were recorded. The frequency histograms of materials in models with the maximum *SCORE* values are shown in Figure 6. Important materials were defined to have an occurrence frequency greater than 7 in this work. As shown in Figure 6A, among the materials in the selected HST models, MCC vivapur^®^ type102 is the high-frequency material, which has Cat.1 tabletability according to index *d*. As shown in Figure 6B, among materials in the selected RST models, processed *Radix glycyrrhizae extractlactose* extract is the high-frequency material, which is classified as having Cat.2A tabletability. The compression curves of the two high-frequency materials obtained are plotted in Figure S7 in the Supplementary Materials. The Cat.1 material (MCC vivapur^®^ type102) with excellent tabletability could exceed 3 Mpa when the tableting pressure was lower than 100 Mpa. The Cat.2A material (processed *Radix glycyrrhizae* extract) had good tabletability when the pressure was above 100 Mpa.

**Figure 7:**
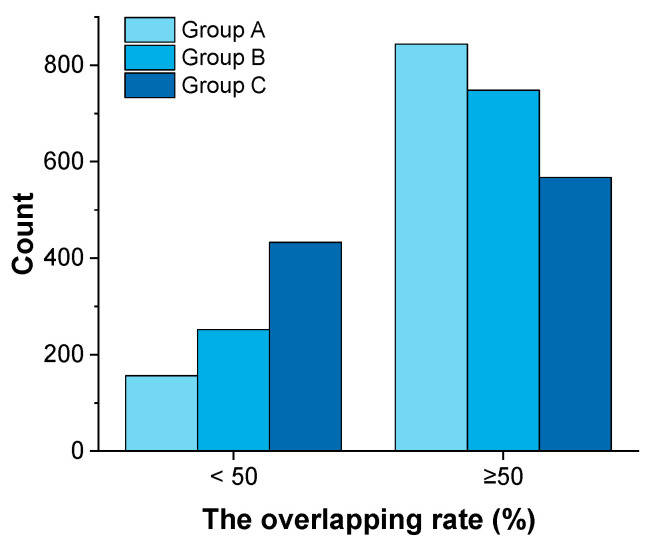
The histogram of overlapping area rate for 3 groups of datasets. Materials in 3 datasets were constructed as follows: (A) two important materials and three hierarchically sampled materials; (B) two important materials and three randomly sampled materials; (C) five randomly sampled materials without two important materials.

To study the effect of two identified important materials on constructing the material library and the resulting model performance, the material library with sample size 5 was investigated. Three kinds of material libraries were constructed: (A) two important materials and three hierarchically sampled materials; (B) two important materials and three randomly sampled materials; (C) five randomly sampled materials without two important materials. For each kind of material library, 1000 rounds of sampling were carried out. After that, PLS models were established, and the rate of overlapping area was calculated. The results are shown in Figure 7, and details are listed in Table S4. In Group C, the frequency count was highest when the overlapping area rate was less than 50%. Compared with Group C models, over 70% of the models in Group B had an overlapping area rate higher than 50%, and over 80% of the models in Group A had an overlapping area rate larger than 50%. These results proved that important materials could ensure information richness and enable better model performance. The overlapping area rate can be used as an indicator to quickly judge the diversity of samples in the sampling subset. The sample set obtained by HST was helpful for ensuring the representativeness and diversity of samples in the training set. The information richness is as important as the data volume, which would challenge the idea that “bigger is better” [55].

## 4. Conclusions

This paper proposes the following assumption: Is there a minimum sample size to develop an acceptable predictive model? Based on the primary material library including 45 materials, both the hierarchical sampling supervised by index *d* and the random sampling method were used to obtain subsets with different sample sizes and material compositions. Then, the HST and RST predictive models were compared and analyzed from the aspects of cross-validation and external validation performance. The differences between performances were assessed in different scenarios. The selection conditions of sample size and type were further summarized with the index *SCORE* and the overlapping area rate. Using this approach, the sample size requirements of a material library were summarized. (1) A minimum dataset with 15 or 20 representative and diverse materials was feasible to develop an acceptable predictive model; (2) the supervised sampling method guided by index *d* was implemented to make the development phase more effective than random sampling; (3) the important materials, such as microcrystalline cellulose and processed *Radix glycyrrhizae* extract, could be considered in building an initial material library.

Collecting material property data may face many challenges, such as tedious testing, high time investment, high labor costs, and material consumption. The data size is an important and common factor affecting the availability of a material library. This paper only applied a material library comprising 45 single materials to investigate the change in model performance, and the conclusions drawn require further validation through the formulation of mixtures and a more comprehensive database. Furthermore, while only index *d* was employed as a classification indicator, more sophisticated classification systems could be considered in future research.

## Figures and Tables

**Table 1:** The sizes and applications of material libraries reported in 2018~2023.

No.	Number of Samples	Number of Material Attributes	Year	Application Area	Reference
1	20	30	2018	Find surrogate materials for pharmaceutical process development.	[1]
2	55	Over 100	2018	Build predictive models for in silico process.	[8]
3	41	8	2019	Develop a direct compression decision-making tool to accelerate materials’ screening.	[9]
4	15	25	2019	Predict the volumetric and gravimetric feeding behavior of a low-feed-rate feeder.	[10]
5	130	18	2019	Develop a compression behavior classification system for direct compression.	[4]
6	20	32	2019	Study the effect of tracer material properties on the residence time distribution of continuous powder-blending operations.	[11]
7	20	44	2019	Evaluate material performance on a loss-in-weight feeder.	[12]
8	111	22	2019	Develop a compression behavior classification system for roll compaction.	[3]
9	12	18	2019	Analyze the effect of material attributes on the dissolution profile of the matrix tablet.	[13]
10	10	30	2020	Analyze the impact of material attributes on the performance of an auger dosing process.	[7]
11	13	44	2021	Predict feeding performance based on material properties.	[14]
12	81	28	2021	Develop machine learning models by linking material properties and direct compression tablet properties.	[15]
13	27	48	2021	Develop a TPLS model for the twin-screw wet granulation process and formulation development.	[16]
14	56	18	2021	Develop a formulation process quality model for high-shear wet granulation.	[17]
15	12	44	2022	Analyze the impact of material attributes on the gravimetric feeding process.	[18]
16	14	55	2022	Develop a TPLS model for the direct compression process and formulation design.	[19]
17	32	19	2022	Develop a PCA model to recognize the highest amount of variability in physical powder properties.	[20]
18	30	19	2022	Develop a tabletability change classification system for high-shear wet granulation and tableting.	[21]
19	15	14	2023	Analyze the impact of material attributes on direct compression extended-release formulations.	[22]
20	31	18	2023	Develop a tabletability change classification system for roll compaction, dry granulation, and tableting.	[23]

**Table 2:** Overview of powder characterization techniques, corresponding descriptors, and abbreviations.

Property	Characterization Technique	Descriptor	Abbreviation
Powder properties	Powder density test	Bulk and tapped density	*ρ*_b_ and *ρ*_t_
		Ture density and porosity	*D*_t_ and *ε*
		Carr’s index	*IC*
		Inter-particle porosity	*Ie*
		Hausner ratio	*IH*
	Diametrical crushing test under maximum compression pressure	Cohesion index	*Icd*
	Flow through an orifice	Angle of repose	*AOR*/*α*
		Flow time	*t*″
	Rapid moisture test	Moisture content	*HR*%
	Moisture sorption	Water uptake at 76% (±2%) of the relative humidity	*H*%
	Laser diffraction	10, 50, and 90% cumulative undersize of volumetric particle size distribution	*D*_10_, *D*_50_, *D*_90_
		Width and span of volumetric particle size distribution	*Span*
		Percentage of particles measuring <50 μm	%*Pf*
		Homogeneity index	*Iθ*
Compression descriptor	The *Kawakita* model	$\frac{P}{C}=\frac{P}{a}+\frac{1}{ab}$	*a*, *ab*, *b*^−1^
	The *Heckel* model	$\ln\left(\frac{1}{\varepsilon }\right)=kP+A$ $P_{y}=\frac{1}{k}$	*P*_y_
	The *Gurnham* model	$\varepsilon =-\frac{1}{K}ln(\frac{P}{P_{0}})$	*K*
	The *Ryshkewitch–Duckworth* model	$TS=exp(-k_{b}\varepsilon )$	*k*_b_
	The *Shapiro* model	$\ln\left(\varepsilon \right)=\ln\left(\varepsilon_{0}\right)-kP-f\times P^{0.5}$	*f*
	The *Power* model	$TS=dP^{g}$	*d*, *g*
Tablet Mechanical property	Diametrical crushing test	Tensile strength	TS

**Table 3:** The factors and levels of the validation design for arranging 24 binary mixtures.

Factors	Levels			
Composition A	MCC PH102	Lactose Tablettose^®^ 80		
Composition B	*Stellariae Radix* extract	*Radix Rehmanniae* Preparata extract	*Rhizoma Alismatis* extract	*Flos Farfarae* extract
Ratio of A to B (*w*/*w*)	1:3	1:2	1:1	

**Table 4:** The maximum and minimum *RMSE* values of training models during cross-validation.

	Max	Min		Max	Min
HST1	1.31	0.41	RST1	1.33	0.19
HST2	1.21	0.52	RST2	1.30	0.28
HST3	1.19	0.60	RST3	1.15	0.48
HST4	1.15	0.71	RST4	1.20	0.55
HST5	1.14	0.77	RST5	1.20	0.71
HST6	1.14	0.80	RST6	1.14	0.76
HST7	1.10	0.88	RST7	1.12	0.83
HST8	1.08	0.93	RST8	1.09	0.91
HST9	1.04	1.03	RST9	1.04	1.03

**Table 5:** The maximum and minimum $R^{2}p$ values of models during external validation.

	Max	Min		Max	Min
HST1	0.94	0.00	RST1	0.94	0.00
HST2	0.95	0.05	RST2	0.95	0.01
HST3	0.95	0.33	RST3	0.95	0.16
HST4	0.95	0.50	RST4	0.95	0.45
HST5	0.95	0.79	RST5	0.95	0.72
HST6	0.95	0.82	RST6	0.95	0.74
HST7	0.95	0.85	RST7	0.95	0.84
HST8	0.94	0.91	RST8	0.94	0.88
HST9	0.93	0.93	RST9	0.93	0.93

**Table 6:** The maximum and minimum of *RMSEp* values of models during external validation.

	Max	Min		Max	Min
HST1	9.70	0.49	RST1	41.56	0.52
HST2	4.09	0.41	RST2	4.30	0.48
HST3	2.88	0.45	RST3	4.12	0.45
HST4	2.30	0.49	RST4	2.55	0.45
HST5	1.68	0.52	RST5	2.39	0.47
HST6	1.44	0.46	RST6	1.74	0.51
HST7	1.30	0.56	RST7	1.30	0.58
HST8	1.20	0.65	RST8	1.23	0.66
HST9	0.97	0.97	RST9	0.97	0.97

## Data Availability

The data presented in this study are available in the Supplementary Materials.

## Supplementary Materials

The following supporting information can be downloaded at: https://www.mdpi.com/article/10.3390/pharmaceutics16020242/s1, Supplementary Materials S1: Figure S1: The boxplots of 45 materials physical properties (A) bulk density; (B) *Icd*; (C) *AOR*; (D) *D*_50_. Figure S2: The histograms of root mean square error from cross-validation. Figure S3: The histograms of correlation coefficient from external validation. Figure S4: The histograms of root mean square error from external validation. Figure S5: The scatter plots of prediction performance of hierarchical sampling models screened by 3 evaluation indices. Figure S6: The scatter plots of prediction performance of random sampling models screened by 3 evaluation indices. Figure S7: The compression curves of identified important materials. Table S1: The information of material libraries reported in 2018~2023. Table S3: The maximum and minimum of *R*^2^ values of models during cross validation. Table S4: The PLSR performance and overlapping area rate of 3 models. Supplementary Materials S2: Table S2: The information of 45 materials including names, abbreviations, batch numbers suppliers and physical attributes. References [1,3,4,7,8,9,10,11,12,13,14,15,16,17,18,19,20,21,22,23] are cited in the supplementary materials.

## Abbreviations

CBCS	Compression behavior classification system
iTCM	intelligent Traditional Chinese Medicine
API	Active pharmaceutical ingredient
DC	Direct compression
TS	Tensile strength
AOR	Angle of repose
PCA	Principal component analysis
PLS	Partial least squares regression
ST	Sampling training dataset
HST	Hierarchical sampling training datasets
RST	Random sampling training datasets
Max	Maximum
Min	Minimum
*R* ^2^	Correlation coefficient
RMSE	Root mean square error
MAPE	Mean absolute error percentage
NPP	Natural Production Powder
PC	Principal component
LV	Latent variable
