# Supplementary material for: Sample Size Requirements of a Pharmaceutical Material Library: A Case in Predicting Direct Compression Tablet Tensile Strength by Latent Variable Modeling
Source: Pharmaceutics. 2024 Feb 7;16(2):242. doi: 10.3390/pharmaceutics16020242 (PMC10893091; doi:10.3390/pharmaceutics16020242)
Supplement: Supplementary file 1 [file pharmaceutics-16-00242-s001.zip › Supplementary materials S1.pdf]

Supplementary Materials: Figure S1, Figure S2, Figure S3, Figure S4, Figure S5, Figure S6 and Figure S7, Table S1, Table S3 and Table S4.

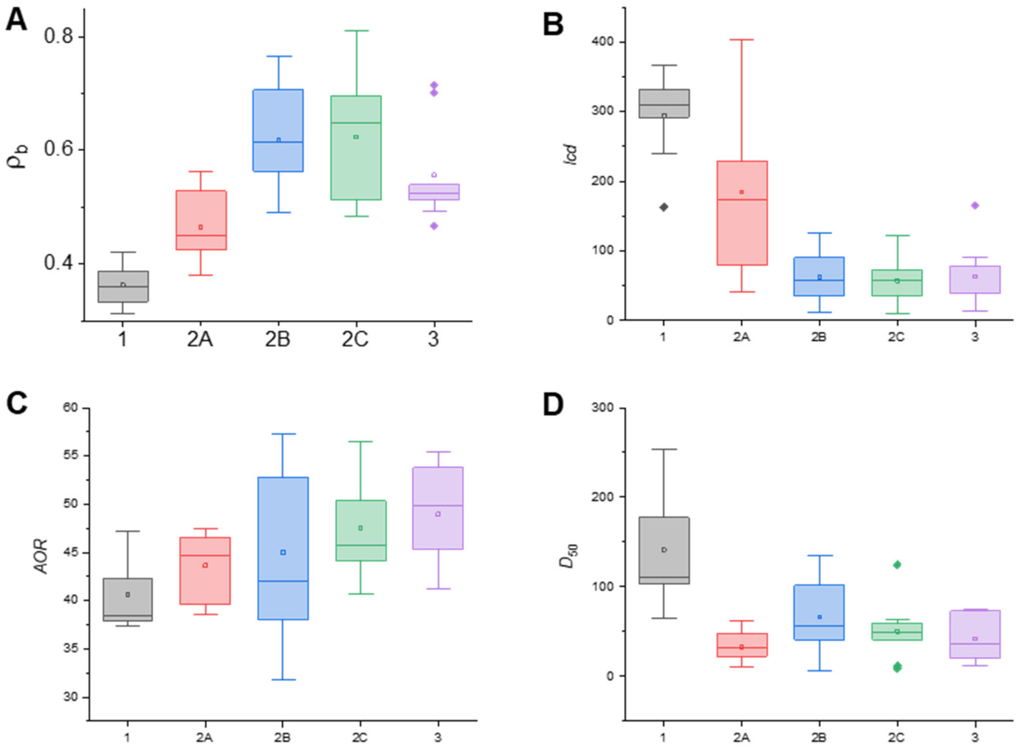

Figure S1. The boxplots of 45 materials related to 4 properties (A) bulk density; (B) *Icd*; (C) *AOR*; (D) D_{50}.

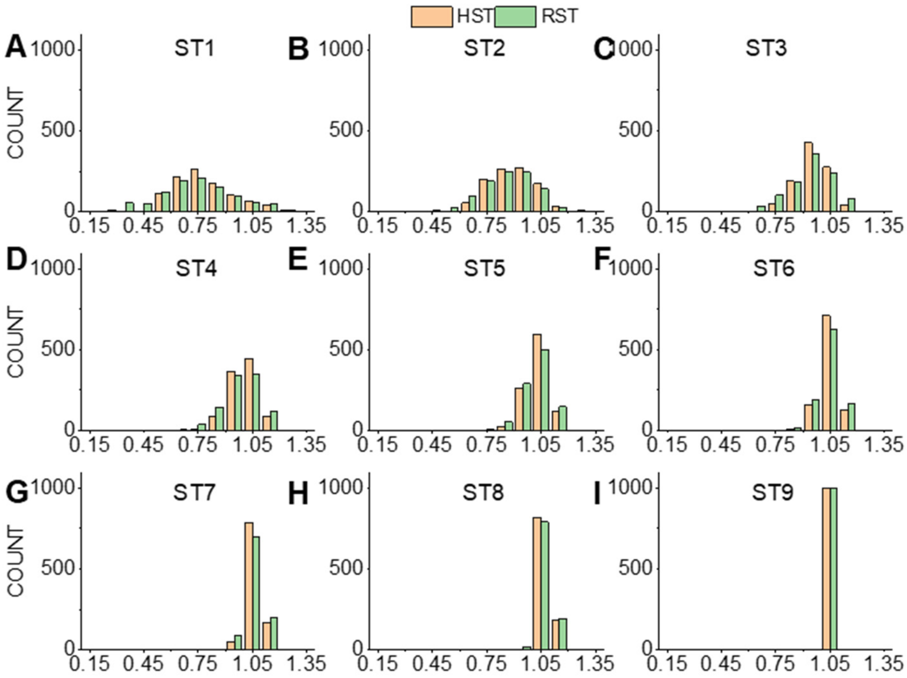

Figure S2. The histograms of root mean square error from cross-validation. Each subgraph (A)~(I) represents a group of few-shot sampling dataset *i* models (sample size=5×*i*, *i* =1~9). The orange color represents hierarchical sampling models and the green color represents random sampling models.

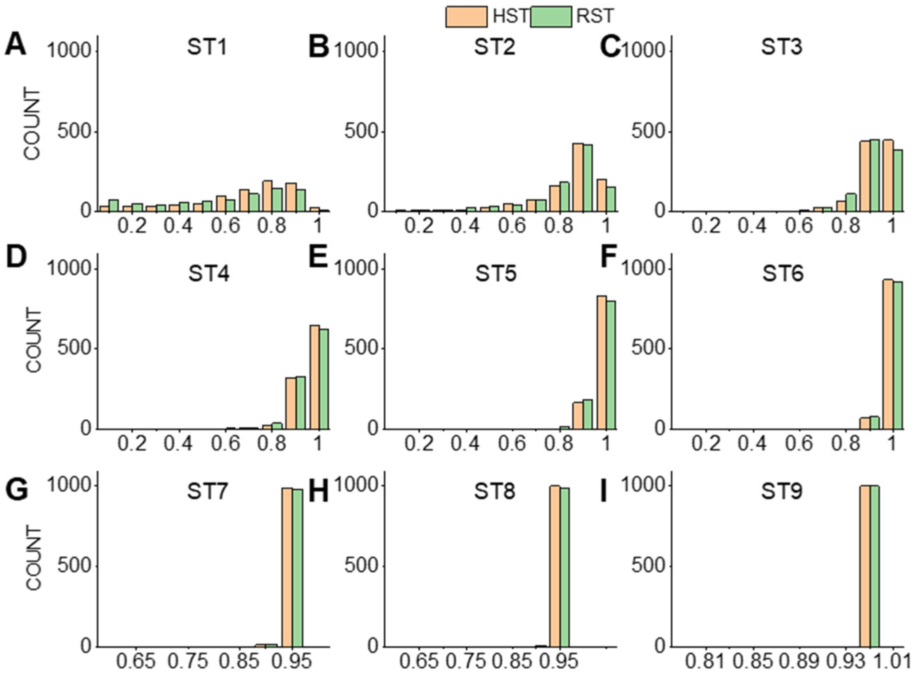

Figure S3. The histograms of correlation coefficient from external validation. Each subgraph (A)~(I) represents a group of few-shot sampling dataset *i* models (sample size=5×*i*, *i* =1~9). The orange

color represents hierarchical sampling models and the green color represents random sampling models.

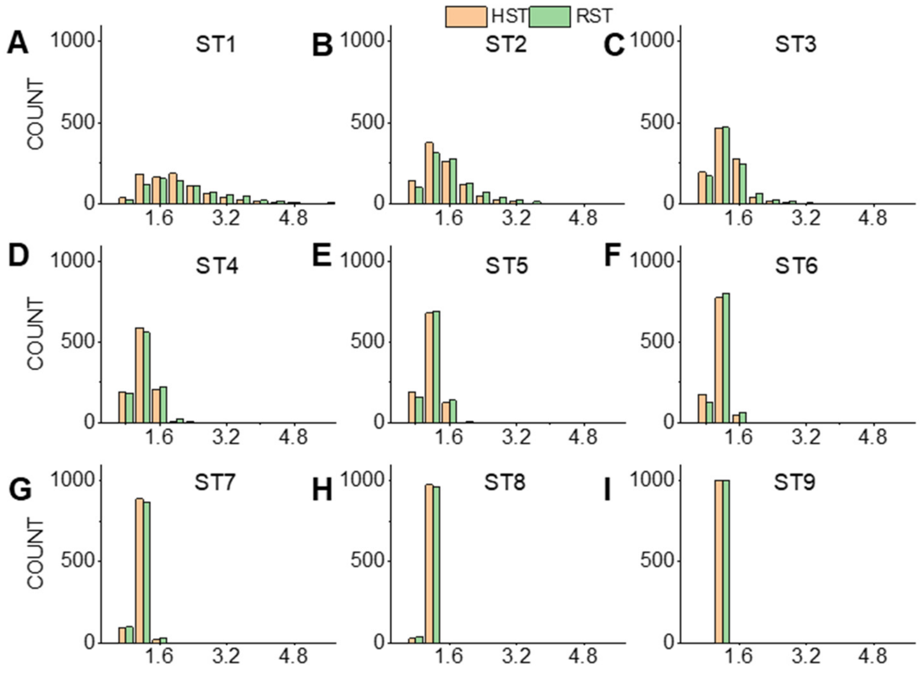

Figure S4. The histograms of root mean square error from external validation. Each subgraph (A)~(I) represents a group of few-shot sampling dataset *i* models (sample size=5×*i*, *i*=1~9). The orange color represents hierarchical sampling models and the green color represents random sampling models.

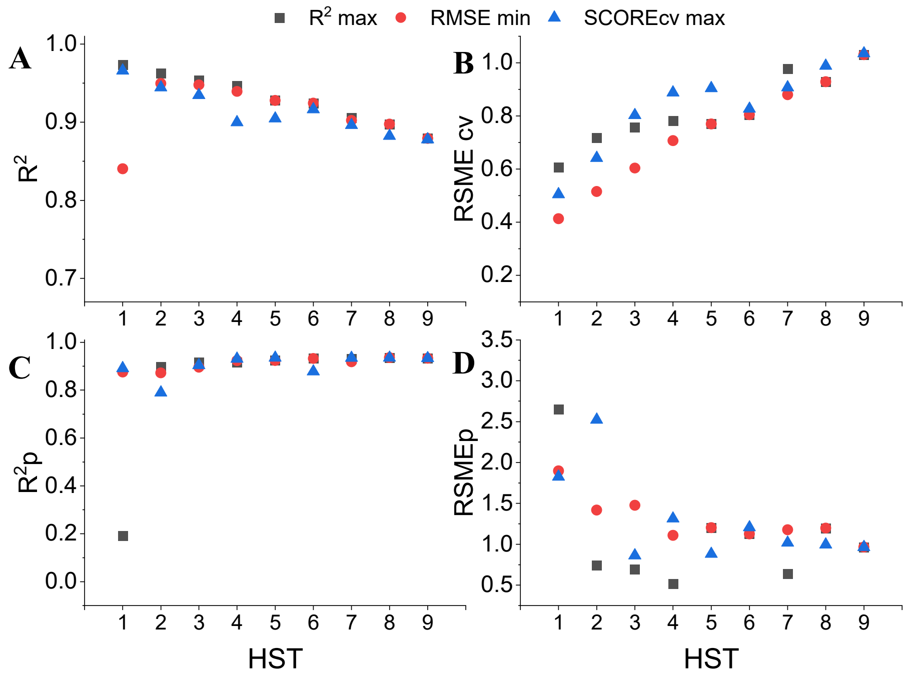

Figure S5. The scatter plots of prediction performance in hierarchical sampling models screened by 3 evaluation indices. Subgraph (A)~(D) represent 4 prediction performance indicators of models, R^2, *RMSE*, R^2p and *RMSEp*, respectively. Black color represents R^2-max which is one of screening indices, red color represents *RMSE*-min, and bule color represents *SCOREcv*-max.

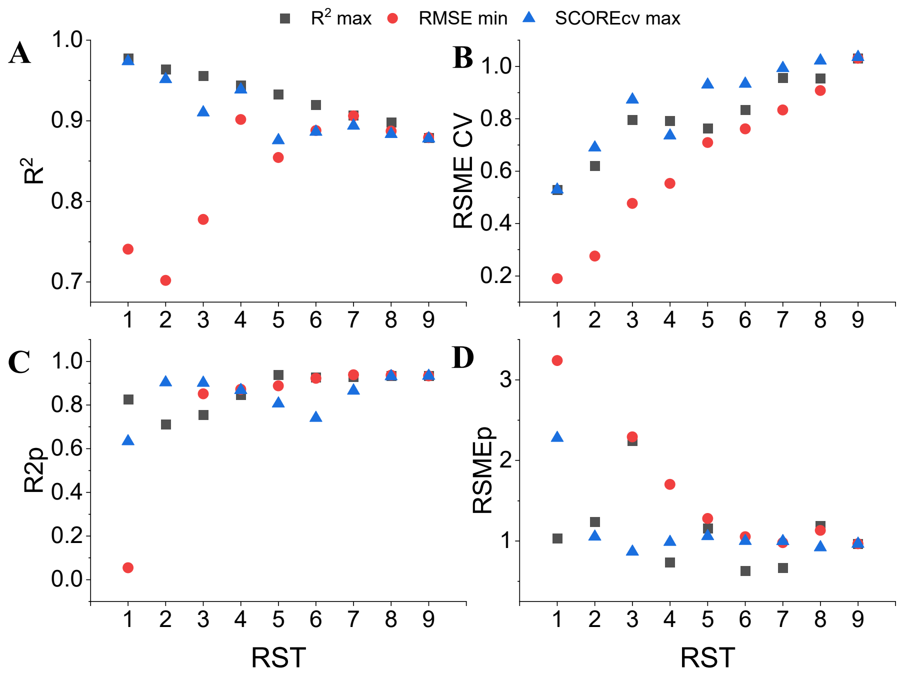

Figure S6. The scatter plots of prediction performance in random sampling models screened by 3 evaluation indices. Subgraph (A)~(D) represent 4 prediction performance indicators of models, R^2, *RMSE*, R^2p and *RMSEp*, respectively. Black color represents R^2-max which is one of screening indices, red color represents *RMSE*-min, and bule color represents *SCOREcv*-max

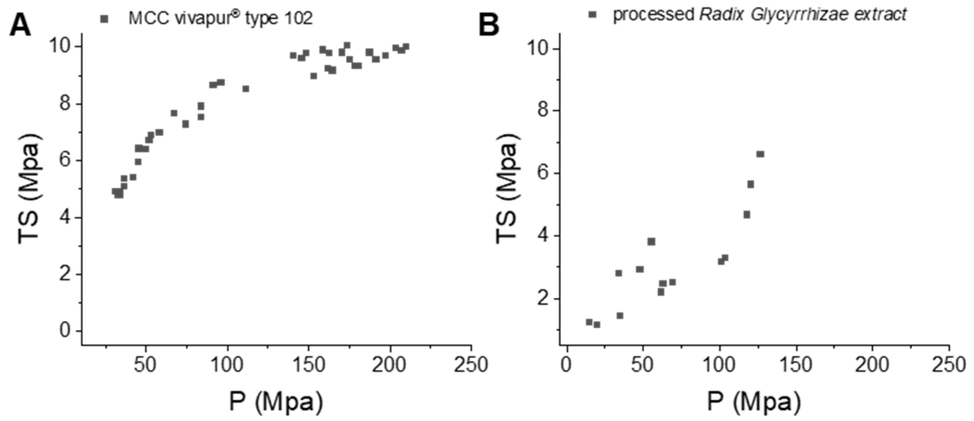

Figure S7. The compression curves of high frequency materials under the maximum *SCORE* value (A) MCC vivapur®type102 in hierarchical sampling models; (B) processed *Radix Glycyrrhizae extract* in random sampling models.

Supplementary Materials: Table S1, Table S3, Table S4

Table S1. The information of material libraries information reported in the 2018~2023.

No.	Sample size	Material attributes	Application area	Sample name	Material attribute test	Years	References
1	20	30	Find surrogate materials for pharmaceutical process development	API, Cellulose, Croscarmellose Sodium, Crospovidone, Magnesium Stearate, Starch, etc.	Density, particle size, flowability (Cohesion, Unconfined Yield Stress, Major Principal Stress, Main Consolidation Stress, and Flow Function Coefficient, Angle of Internal Friction), etc.	2018	[1]
2	55	Over 100	Be used as the basis to build predictive models for *in silico* process.	API, Cellulose, Lactose, Mannitol, Starch, Copovidone, HPMC, Co-processed excipients, Magnesium Stearate, Talc, Crospovidone, etc.	Density, compressibility, moisture sorption, permeablity and fluidization, powder flow, wall fraction, particle size and shape, surface area, porosity, electrostatic charge, etc.	2018	[8]
3	41	8	Develop a DC decision-making tool to accelerate materials screening.	API, Lactose, Microcrystalline cellulose, Hydroxypropyl methylcellulose, Magnesium stearate.	Particle size distribution (D10, D50, D90), specific surface area (SSA), bulk density (BD), tapped density (TBD), Carr's index (CARR) and Hausner ratio (HR), etc.	2019	[9]
4	15	25	Predict the volumetric and gravimetric feeding behavior of a low feed rate feeder	API, Cellulose, Lactose, Dibasic calcium phosphate, Crospovidone, Pre-gelatinized starch, Magnesium stearate.	Density, particle size, flowability, charge density.	2019	[10]

5	130	18	Develop a compression behavior classification system for DC.	NPP, Cellulose, Lactose, Mannitol, Starch, Copovidone, HPMC, Magnesium Stearate, Talc, Carboxy methyl starch sodium, Polyvinylpolypyrrolidone, Dextrin, etc.	Density, compressibility, flowability, moisture sorption, particle size, compression descriptor, etc.	2019	[4]
6	20	32	Study the effect of tracer material properties on the residence time distribution of continuous powder blending operations	API, Cellulose, Croscarmellose Sodium, Crospovidone, Magnesium Stearate, Starch, etc.	Density, particle size, flowability (Cohesion, Unconfined Yield Stress, Major Principal Stress, Main Consolidation Stress, and Flow Function Coefficient, Angle of Internal Friction)	2019	[11]
7	20	44	Evaluate material performance on a loss-in-weight feeder.	API, Cellulose, Croscarmellose Sodium, Crospovidone, povidone, sodium stearyl fumarate, magnesium stearate, calcium phosphate anhydrous, hypromellose, etc.	Particle size distribution, flowaibility, compressibility, permeability, etc.	2019	[12]
8	111	22	Develop a compression behavior classification system for roll compaction.	NPP, Cellulose, Lactose, Mannitol, Starch, Copovidone, HPMC, Magnesium Stearate, Talc, Carboxy methyl starch sodium, Polyvinylpolypyrrolidone, croscarmellose sodium, etc.	Density, particle size, flowability, compressibility, stability and texture, etc.	2019	[3]
9	12	18	Analyze the effect of the material attributes on the dissolution profile of the matrix tablet.	NPP, HPMC	Density, compressibility, flowability, moisture sorption, particle size, etc.	2019	[13]

10	10	30	Analyze the impact of material attributes on the performance of an auger dosing process.	API, cellulose, lactose, starch, ascorbic acid, magnesium stearate, etc.	Density, particle size, mositure, compressibility, flowability, shear properties, dynamic properties, etc.	2020	[7]
11	13	44	Predict the feeding performance based on material properties.	API, Cellulose, Lactose, starch, etc.	Density, compressibility, moisture sorption, permeablity and fluidization, powder flow, wall fraction, particle size and shape, surface area, porosity, electrostatic charge.	2021	[14]
12	81	28	Develop machine learning models between material properties and DC tablet properties.	API, cellulose,magnesium stearate, etc.	Density, compressibility, moisture sorption, in-die elastic recovery, molecular weight, the partition coefficient, solubility, hygroscopicity, WAR, and surface free energy, etc.	2021	[15]
13	27	48	Develop a TPLS model for twin-screw wet granulation process and formulation development	API, cellulose, lactose, starch, mannitol, hydroxypropyl methylcellulose, polyvinylpyrrolidone, etc.	Density, particle size, flowability, powder elasticity and plasticity, powder rheology, charge densitym, specific surface area, solubility, dissolution rate, sorption properties, contact angle, etc.	2021	[16]
14	56	18	Develop a formulation-process-quality model for high shear wet granulation	API, cellulose, lactose, starch, PVP, etc.	Density, particle size, flowability, mositure, etc.	2021	[17]

15	12	44	Analyze the impact of material attributes on gravimetric feeding process.	API, cellulose, lactose, mannitol, MgSt, etc.	Density, compressibility, moisture sorption, permeablity and fluidization, powder flow, wall fraction, particle size and shape, surface area, porosity, electrostatic charge.	2022	[18]
16	14	55	Develop a TPLS model for DC process and formulation	API, cellulose, lactose, MgSt, mannitol, croscarmellose, dibasic calcium phosphate, colloidal silicon dioxide, etc.	Density, compressibility, moisture sorption, powder rheometer, powder flow, wall fraction, particle size and shape, surface area, porosity, static image, charge density, etc.	2022	[19]
17	32	19	Develop a PCA model to recognize the highest amount of variability in physical powder properties.	API, cellulose, lactose, MgSt, mannitol, croscarmellose, dibasic calcium phosphate, colloidal silicon dioxide, Co-processed excipients, PVP, etc.	Density, compressibility, moisture sorption, powder rheometer, powder flow, wall fraction, particle size and shape, surface area, porosity, static image, charge density, etc.	2022	[20]
18	30	19	Develop a tabletability change classification system for high shear wet granulation.	NPP, cellulose, lactose, starch, dextrin, mannitol, calcium phosphate, dibasic calcium phosphate, dibasic calcium phosphate anhydrous, etc.	Density, compressibility, flowability, moisture sorption, particle size, compression descriptor, tabletability change index, etc.	2022	[21]
19	15	14	Develop a tabletability change classification system under roll compaction granulation.	Cellulose, lactose, MgSt, mannitol, HPMC, Polyvinyl alcohol, Poly(ethylene)oxide, Maltodextrin, etc.	Density, particle size, flowability, compressibility, etc.	2023	[22]

20	31	18	Analyze the impact of material attributes on direct compressible extended release formulations.	NPP, cellulose, lactose, hydroxypropyl methylcellulose (HPMC), low-substituted hydroxypropyl cellulose (L-HPC), croscarmellose sodium (CCNa), corn starch, D-sorbitol, DCP and sodium bicarbonate, etc.	Density, compressibility, flowability, moisture sorption, particle size, compression descriptor, tabletability change index, etc.	2023	[23]

Table S3. The maximum and minimum of R^2 values of models during cross validation.

	Max	Min
HST1	0.97	0.62
HST2	0.96	0.62
HST3	0.95	0.74
HST4	0.95	0.81
HST5	0.93	0.81
HST6	0.92	0.83
HST7	0.91	0.84
HST8	0.90	0.86
HST9	0.88	0.88

	Max	Min
RST1	0.98	0.59
RST2	0.96	0.49
RST3	0.96	0.51
RST4	0.94	0.60
RST5	0.93	0.71
RST6	0.92	0.77
RST7	0.91	0.78
RST8	0.90	0.83
RST9	0.88	0.88

Table S4. The PLSR performance and overlapping area rate of 3 models. Materials in 3 datasets were constructed by (A) two important materials and three hierarchically sampled materials; (B) two important materials and three randomly sampled materials; (C) five randomly sampled materials without 2 important materials.

	Group A	Group B	Group C
R^2	0.91~0.96	0.79~0.96	0.55~0.98
RMSE	0.68~1.11	0.62~1.23	0.08~1.31
SCOREcv	0.28~3.25	0.26~7.61	0.21~6.51
R^2p	0.43~0.94	0~0.94	0~0.93
RMSEp	0.46~1.5	0.47~3.96	0.6~6.73
SCOREp	1.68~8.42	-0.08~7.56	-1.18~7.05
Overlapping area rate	18~100%	5~100%	3~100%
